# Validation of Quantitative Measurements in Cardiovascular Imaging

**DOI:** 10.1155/2015/321623

**Published:** 2015-07-12

**Authors:** Peter M. A. van Ooijen, Marco Francone, Joachim Lotz, Volker Rasche

**Affiliations:** ^1^Department of Radiology, University Medical Center Groningen, University of Groningen, Groningen, Netherlands; ^2^Department of Radiological Oncological and Pathological Sciences, Sapienza University of Rome, Rome, Italy; ^3^University Medical Center Göttungen, Institute of Clinical and Interventional Radiology, Göttingen, Germany; ^4^University of Ulm, Ulm, Germany

## 1. Introduction

Recent developments in cardiovascular imaging have shown a trend towards quantitative measurement of findings. These quantitative measurements could eventually be the basis to obtain quantitative imaging biomarkers that could be used to predict, diagnose, and treat disease. However, the first step to achieve quantitative imaging biomarkers is to establish proper validation of these quantitative measurements to ensure that the numbers obtained from the imaging data are reliable, reproducible, accurate, and clinically useful. This process involves the proper standardization and validation of all steps in the imaging process from acquisition, preprocessing, and postprocessing to reading and reporting. With the papers published in this special issue we hope to increase the evidence required to achieve quantitative imaging biomarkers in cardiovascular imaging by validation of those quantitative measurements in phantoms, animal models, and clinical data.

## 2. Modeling and Phantom Development

To allow proper validation the use of patient or volunteer data is often nonoptimal because of the lack of a gold-standard that can be used to validate the quantification. This often results in the use of other modalities or manual expert reading to validate against. One way of solving this issue could be the implementation of a phantom that can be used to perform the validation. However, a shortcoming of these phantoms is often their lack of realism with respect to anatomy and function. G. J. Pelgrim et al. describe in their contribution how they implemented an ex vivo, perfused porcine heart model for myocardial perfusion CT imaging. These kinds of developments where animal organs are used as basis for a phantom could provide a more real-life environment while also providing the possibility of obtaining the gold standard by additional measurements, controlled experiments, and pathological evaluation.

## 3. Acquisition Technique and Protocol

Acquisition techniques and protocols are vendor specific, resulting in a variation in measured values for the same morphological or functional parameters. To overcome these differences, standardization and calibration of acquisition systems and protocols could be part of the solution.

For example, a review by G. Varetto et al. on the value of contrast enhanced ultrasound (CEUS) for vulnerable plaque analysis. These kinds of reviews can provide valuable information on the validity of acquisition techniques and allow a discussion of the possibility of generalized acquisition protocols with high accuracy and reliability to allow a more standardized quantification. One of the aspects revealed by this study that although the reliability of the acquisition technique is high, a shared, user-friendly protocol of imaging analysis is not available yet although this would be mandatory to allow comparison of results.

Patient cooperation can also be an issue in the acquisition of data that can be used for quantitative evaluation. Y. Zhu et al. show that the accuracy and reproducibility of the current technique for right ventricular evaluation using 2D breath-hold MR can be compromised by slice misregistration or requirement to use a larger slice gap. This is mainly caused by the inability of the patients to hold their breath consistently during the acquisition. To overcome these problems 3D scan protocols are developed that are hampered by the fact that they require a long breath-hold. They therefore propose, in their paper, the use of a free-breathing 3D imaging acquisition to tackle these problems while retaining a dataset of proper quality to allow quantitative measurement of RV function.

## 4. Postprocessing Algorithms and Validation

Postprocessing techniques are applied in all imaging modalities to obtain quantitative information. However, there is usually no standardization available and all research groups and vendors have their own algorithms and approach. Part of the solution is in the automation of algorithms, thus eliminating the observer influence. Another part is the extensive cross-validation of different algorithms and approaches.

Segmentation of the left ventricle for the determination of left ventricular function has been an area of research for quite some time already. The literature has shown an increasing reproducibility and accuracy of algorithms used for this segmentation although many still rely on either manual segmentation or manual correction of automatically generated contours. One of the main problems in the segmentation of the left ventricle is its complex motion during the cardiac cycle. Of this the long-axis motion is often not taken into account. The study by J. Tufvesson et al. presented in this special issue aims to develop and validate an automatic postprocessing algorithm for time-resolved segmentation, covering the whole LV, including basal slices affected by long-axis motion. They show minor differences between manual segmentation and their automatic segmentation resulting in an algorithm that is very well applicable in clinical practice.

Another example of automation in segmentation of areas of interest is the work by V. Tuncay et al. who describe a newly developed semiautomatic segmentation technique to determine the Aortic Valve Area in MDCT datasets. They show that automation of this measurement provides accurate, more reproducible, and faster measurement. Validation was done by comparing both to manual expert segmentation and to measurements obtained with a more established modality (Transthoracic Echocardiography) performed in the same patient.

The importance of proper validation and calibration of measurements is also demonstrated in the paper by I. Carbone et al. They studied the suitability of several skeletal muscles as reference regions for calculating the T2-SI-ratio for semiautomatic quantification of the extent of myocardial edema. In result it was shown that using the serratus anterior is the most reliable and reproducible muscle for measuring the extent of myocardial edema.

P. Maheshwari et al. show in their review paper that although a measurement such as the modified Myocardial Performance Index (Mod-MPI) is well-established, standardization can still be lacking resulting in varying outcomes between different operators. They therefore propose an automated measurement of the Mod-MPI to allow its proper implementation into clinical use.

*Peter M. A. van Ooijen* *Marco Francone* *Joachim Lotz* *Volker Rasche*

